# Involvement of Cytochrome P450 in Pentachlorophenol Transformation in a White Rot Fungus *Phanerochaete chrysosporium*

**DOI:** 10.1371/journal.pone.0045887

**Published:** 2012-09-20

**Authors:** Daliang Ning, Hui Wang

**Affiliations:** State Key Joint Laboratory on Environment Simulation and Pollution Control, School of Environment, Tsinghua University, Beijing, People's Republic of China; Missouri University of Science and Technology, United States of America

## Abstract

The occurrence of cytochrome P450 and P450-mediated pentachlorophenol oxidation in a white rot fungus *Phanerochaete chrysosporium* was demonstrated in this study. The carbon monoxide difference spectra indicated induction of P450 (103±13 pmol P450 per mg protein in the microsomal fraction) by pentachlorophenol. The pentachlorophenol oxidation by the microsomal P450 was NADPH-dependent at a rate of 19.0±1.2 pmol min^−1^ (mg protein)^−1^, which led to formation of tetrachlorohydroquinone and was significantly inhibited by piperonyl butoxide (a P450 inhibitor). Tetrachlorohydroquinone was also found in the cultures, while the extracellular ligninases which were reported to be involved in tetrachlorohydroquinone formation were undetectable. The formation of tetrachlorohydroquinone was not detectable in the cultures added with either piperonyl butoxide or cycloheximide (an inhibitor of *de novo* protein synthesis). These results revealed the pentachlorophenol oxidation by induced P450 in the fungus, and it should be the first time that P450-mediated pentachlorophenol oxidation was demonstrated in a microorganism. Furthermore, the addition of the P450 inhibitor to the cultures led to obvious increase of pentachlorophenol, suggesting that the relationship between P450 and pentachlorophenol methylation is worthy of further research.

## Introduction

Pentachlorophenol (PCP) has been widely applied for decades around the world, as a fungicide (mainly as wood preservative), herbicide, defoliant and detergent supplement in soaps [1]. In consequence, PCP and its residues can be found today as a group of major pollutants in many terrestrial and aquatic ecosystems [2], [3], and become widespread in humans and other living organisms, including those without direct exposure [4]. In addition to obvious acute effects, PCP can cause birth defects, chromosome abnormalities, blood disorders, nerve damage [4], cancer (confirmed in animal and probable in human) [5] and endocrine disruption [6]. Because of such high toxicity and persistence in the environment, there is now a complete ban on PCP production within the European Union (EU). Moreover, many countries and regions, such as China, USA and EU, classified PCP as a priority pollutant and have recommended restricted use to minimize its impact [7], [8].

Biotransformation by microorganisms is an important fate of PCP in the environment and contaminated sites. The white rot fungi, which can decompose lignin, can effectively transform PCP and were widely studied for the treatment of PCP-contaminated soil [9], [10] and wastewater [11]. Dechlorination and methylation were the two major pathways of PCP biotransformation and both led to detoxification of PCP. Aerobic dechlorination of PCP is known to be catalyzed by hydroxylase (a flavoprotein in *Flavobacterium sp*.) [12] and various peroxidases, such as laccase [13], lignin peroxidase (LiP), manganese peroxidase (MnP) [14] in the white rot fungi, and horseradish peroxidase [15]. O-methylation of chlorophenols has been reported to be an environmentally significant alternative to the dechlorination [2]. PCP methylation was found in some bacteria [16], and much more common among fungi including the white rot fungi [1], [17].

Cytochrome P450s (P450s) are widely distributed hemoproteins involved in various steps of the biosynthesis of endogenous compounds and in the oxidative detoxification and elimination of many hydrophobic xenobiotics including pollutants, drugs and pesticides [18]. PCP can obviously induce cytochrome P450 (P450) activities in animals and insects [19]–[21], and was reported to be transformed into tetrachlorohydroquinone (TCHQ) by a human P450 (CYP 3A4) [22]. Involvement of P450-type enzyme in PCP oxidation was suspected according to results of inhibitor assay in *Mucor ramosissimus* [1] and spectrometric evidences in *Mycobacterium chlorophenolicum* (formerly *Rhodococcus chlorophenolicus*) [23]. However, P450-mediated oxidation of PCP was still not elucidated in any microorganism.

The white rot fungus *Phanerochaete chrysosporium* has more than 150 P450 genes, which is one of the largest P450 contingents known to date in fungi [24] and the functions of which are of special interest in recent years [25], [26]. In our previous study, we proposed significant induction of *P. chrysosporium* P450 by PCP according to the carbon monoxide (CO) difference spectrum with a peak at 457 nm, and found an increase of PCP degradation with the presence of P450 inhibitor [27]. In this paper, the P450-mediated oxidation was demonstrated to be involved in PCP transformation by this fungus.

## Materials and Methods

### Chemicals

PCP, pentachloroanisole (PCA), and piperonyl butoxide (PB) were obtained from Aldrich, and TCHQ was obtained from Supelco. Cycloheximide (CHI) was obtained from Roche.

### Microorganisms and growth media

*P. chrysosporium* strain BKM-F-1767 (ATCC 24725) was maintained on Difco potato dextrose agar (PDA). Difco potato dextrose broth (PDB, 24 g·l^−1^) was used as nutrient-rich medium.

Inocula were prepared according to the method of Aiken and Logan [28]. Fungi grown for 6 days in a static culture flask were blended and mixed, and then a 0.1-ml aliquot of this suspension was aseptically transferred to a sterile 250-ml flask containing 25 ml of media. The fungi were incubated for 60 h at 37°C.

Then, 0.938 *µ*mol (0.25 mg) of PCP dissolved in 25 *µ*l of acetone was added to each flask to a final concentration of 37.5 *µ*M in the cultures. Two types of controls were prepared, one with sterile culture (autoclaved at 121°C for 20 min) and PCP and the other with intact culture but without PCP. The fungal biomass of the sterile control was approximately equivalent to that in the experimental cultures. All the flasks were incubated at 37°C in darkness (to prevent photooxidation of the PCP). The cultures were routinely examined by microscopy to ensure the absence of bacterial contamination.

### Extraction and detection of PCP and the metabolites

#### Extraction

The flasks were incubation for another 4 days (unless otherwise stated) after addition of PCP.

PCP and the metabolites were extracted according to Reddy et al. [29]. The cultures were added with sodium dithionite to reduce quinone products, acidified with HCl to pH 2, saturated with NaCl, and extracted three times with ethyl acetate. The ethyl acetate extracts were dried over anhydrous sodium sulfate and concentrated with a rotary vacuum evaporator at 35°C. The residues were acetylated with acetic anhydride-pyridine (2∶1) and analyzed by gas chromatography-electron capture detection (GC-ECD) and by GC-mass spectrometry (GC-MS), and then compared with residues from the controls. Quinones were analyzed directly by high-performance liquid chromatography (HPLC) without prior reduction.

#### HPLC analysis

Reverse phase HPLC analysis of metabolites was conducted as described by Reddy et al. [29], with a Hewlett Packard 1100 series liquid chromatography (Hewlett Packard, Palo Alto, CA, USA) equipped with a Lichrospher 100 RP8 column (Agilent Technologies, Santa Clara, CA, USA), 5 µm (250 mm×4.6 mm). The column was eluted with a linear gradient of 0 to 75% acetonitrile in 0.05% phosphoric acid over 15 min, with a flow rate of 1 ml•min^−1^. UV absorbance spectra were determined with the diode array spectrophotometer (Hewlett Packard G1315A) and compared to those of standards. PCP and the quinones were detected at 238 nm and 285 nm, respectively.

#### GC-ECD and GC-MS analysis

GC-ECD analysis was performed on an Agilent 6890N GC with a HP-5 capillary column (30 m×0.25 mm×0.32 µm). GC-MS analysis of derivatized metabolites was performed on a DSQ GC-MS (Thermo Corp., Waltham, MA, USA) with a quadrupole mass filter and a VF-5 MS (Varian, Palo Alto, CA, USA) capillary column (30 m×0.25 mm×0.25 µm), and ran in the electron ionization mode with electron energy of 70 eV. The injector temperature was 200°C. The column was held at 50°C for 2 min, and programmed to rise to 300°C at 10°C•min^−1^, after which the temperature was held isothermally at 300°C for 3 min. The derivatized products were identified by comparing their retention times on chromatography and their mass spectra with derivatized standards, and quantitated by using calibration curves obtained with derivatized standards.

### Preparation of extracellular and microsomal fractions

To prepare extracellular fractions, the culture supernatants were periodically sampled during the incubation. The samples were centrifuged at 9,000 *g* for 10 min and filtered through 0.2 *µ*m-pore-size filter to obtain extracellular fractions.

To prepare microsomal fractions for P450 study, some flasks were incubated for 24 hours after addition of PCP dissolved in acetone. Non-induced and acetone-induced cultures were set up as controls for analysis of P450 induced by PCP. The mycelia were harvested by centrifugation (5000 *g*, 15 min, twice) and the fungal pellets were washed extensively with 0.1 M sodium phosphate buffer (pH 7.5, ice-cold) containing 10 mM EDTA. The microsomal fraction was then prepared according to Masaphy et al. [30]. The pellets were resuspended in 0.1 M sodium phosphate buffer (called buffer B, pH 7.5, ice-cold) containing 1 mM EDTA, 1 mM dithiothreitol (DTT), 0.5 mM phenylmethylsulfonyl fluoride (PMSF) and 20% (v/v) glycerol. Then the cells were disrupted by glass beads (0.4∼0.6 mm, 1 g mycelium in 1 ml buffer B with 1 g glass beads) using a dismembrator (Sartorius, Mikro-Dismembrator S, Germany) over a period of 2 min with 20-s bursts followed by 20-s cooling on ice. The biomass homogenate was centrifuged at 15,000 *g* for 15 min twice to remove cell debris, nuclei and mitochondria. Then the supernatant was centrifuged at 105,000 *g* for 90 min to pellet the microsomal fraction. After washing twice with buffer B, the microsomal fraction was suspended in buffer B and stored at −80°C.

### Enzyme activity assay

#### Cytochrome P450 determination

The P450 contents of microsomal fractions were determined by CO difference spectra as described previously [31]–[33]. Firstly, the total protein concentrations of the microsomal fractions were determined by the Bradford method [34]. The sample was diluted to about 1 mg·ml^−1^ protein with buffer B and then added with 1 mM KCN. Subsequently, the sample was dispensed equally into two cuvettes (1 ml per cuvette) and a baseline spectrum was spectrophotometrically recorded in the range of 400∼500 nm (Shimadzu, UV-2401PC, Japan). One cuvette was then gently gassed with CO at a rate of 3 ml·min^−1^ for 40 s, and the other one was gassed with N_2_ to the same extent. An equal volume (10 µl) of sodium dithionite solution (400 mg·ml^−1^) was accurately added into each cuvette, and then a difference spectrum was recorded. The concentrations of P450 were calculated using the extinction coefficient ε_450_–_490_ value of 91 mM^−1^·cm^−1^ [32]. The P450 contents in microsomal fractions were expressed in pmol P450 per mg protein.

#### Microsomal transformation of PCP

The 1-ml assay solution contained 3 mM MgCl_2_, 1 mM NADPH, 5 mg BSA, microsomal fractions from PCP-induced cells (1∼1.4 mg of protein) and 37.5 µM PCP in 100 mM potassium phosphate buffer (pH 7.5). Three types of control reactions, no-NADPH control, sterile control and the control using microsomal fractions from non-induced cell, were run parallel. The reaction mixtures were incubated at 37°C overnight, and then extracted with ethyl acetate at pH 2, dried over sodium sulfate, evaporated under N_2_ and analyzed by GC-ECD after acetylation. The inhibition of P450 activity was determined with PB (a P450 inhibitor) added to the concentration of 2 mM in the reaction mixture. The decrease in the amounts of PCP transformed and TCHQ formed was measured by GC-ECD.

#### Extracellular peroxidases determination

The activities of LiP, MnP and laccase were determined spectrophotometrically by the method of Tien and Kirk [35], Paszczynski et al. [36] and Niku-Paavola et al. [37]. LiP activity in extracellular fractions was assayed with 3,4-dimethoxybenzyl alcohol as the substrate. MnP activity was assayed with Mn^2+^ as substrate. Laccase activity was assayed with 2,2′-azinobis-(3-ethylbenzthiazoline-6-sulfonate) as substrate. One unit (U) of enzyme oxidizes 1 µmol of the substrate per min in the presence of H_2_O_2_.

#### Extracellular enzyme reaction

Two types of extracellular reaction mixtures were set according to the MnP and LiP reaction mixtures reported by Reddy et al. [29]. The first one contained extracellular fractions (1 ml in the 2-ml mixture), PCP (37.5 µM), MnSO_4_ (0.2 mM), and H_2_O_2_ (0.1 mM) in 50 mM sodium malonate (pH 4.5), and the second one had extracellular fractions (1 ml in the 2-ml mixture), PCP (37.5 µM), veratryl alcohol (0.1 mM), H_2_O_2_ (0.1 mM) in 20 mM sodium succinate (pH 3.0). Reactions were carried out at 25°C for 4 days. Reaction mixtures were extracted with ethyl acetate, evaporated under N_2_ and analyzed by GC-ECD after acetylation as described previously [29].

### Inhibitor experiments

The PDB cultures were incubated for 60 h at 37°C in the dark. Then, PB was added to some flasks to a final concentration of 2 mM, while CHI (1.46 mM) and PMSF (0.5 mM) were added to some other flasks to suppress *de novo* synthesis of proteins and to inhibit protease [38]. After another 60-min incubation, the cultures were added with PCP (37.5 *µ*M), incubated for another 4 days and extracted as described above. Transformation rate of PCP and formation of PCP metabolites were analyzed by GC and compared to those controls without PB or CHI.

The assays were all performed in triplicate and the mean values and standard deviations of the data were presented.

## Results

### Transformation efficiency and metabolites of PCP in the cultures

In the PDB cultures, the fungus was able to transform 18±3% (the mean ± standard deviation of three replicates) of added 37.5 *µ*M PCP in 1 day (6.7±1.1 *µ*M·day^−1^ on average), and 45±3% in 4 days (4.2±0.3 *µ*M·day^−1^ on average). The average transformation efficiency was 1.37±0.23 *µ*mol·day^−1^·g^−1^ (*µ*mol per day per gram dry weight of biomass) in 1 day, and 0.772±0.047 µmol·day^−1^·g^−1^ in 4 days.

**Figure 1:**
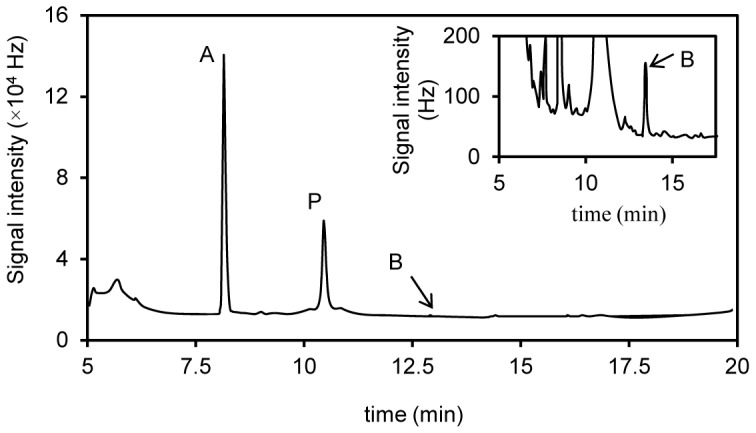
GC-ECD chromatogram of the extracts from the cultures of *Phanerochaete chrysosporium* incubated with pentachlorophenol. After preincubated for 60 h, the 25-ml potato dextrose broth cultures were added with 37.5 *µ*M pentachlorophenol (dissolved in 25 *µ*l acetone), and incubated for 4 days at 37°C in darkness. The cultures were then acidified, extracted and acetylated. P, pentachloroacetoxybenzene. A, pentachloroanisole. B, tetrachloro-1,4-diacetoxybenzene (acetylated tetrachlorohydroquinone). The inner figure shows the peak of compound B by scaling up the vertical axis.

**Figure 2:**
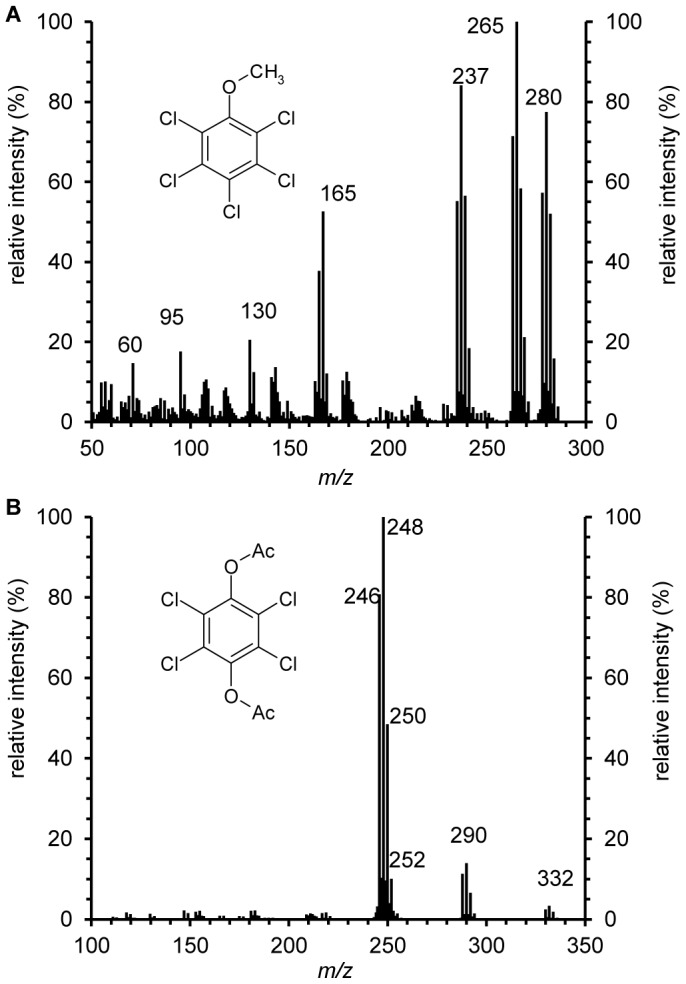
Mass spectra of pentachloroanisole (A) and acetylated tetrachlorohydroquinone (B) produced by *Phanerochaete chrysosporium*. After preincubated for 60 h, the PDB cultures were added with 37.5 *µ*M pentachlorophenol and incubated for 4 days at 37°C in darkness. The metabolites were extracted, acetylated and analyzed by GC-MS.

After concentration of the acetylated extract, the metabolites were analyzed by comparing the GC profiles (Figure 1) with those of acetylated extracts from the control cultures without PCP. The two metabolites were then identified as PCA and TCHQ, according to comparison of the retention times on GC and the mass spectra with derivatized standards (Figure 2). While 16.9±1.2 µM PCP was transformed in the cultures incubated with PCP for 4 days, the concentrations of the products, PCA and TCHQ, were 15.2±1.2 *µ*M and 0.31±0.09 *µ*M respectively. This result indicated that methylation was the major but not the only way of PCP biotransformation by *P. chrysosporium*.

### Role of the extracellular peroxidases

The production of TCHQ indicated PCP oxidation by *P. chrysosporium* which was only reported to be catalyzed by the extracellular ligninases, LiP and MnP [14]. However, the ligninases, LiP, MnP and laccase, were all undetectable in the PDB cultures incubated with PCP for 0, 1, 2, 3 or 4 days. Moreover, neither PCP transformation nor TCHQ were detectable in the reaction mixtures of extracellular fractions from the PDB cultures. These results ruled out detectable involvement of the extracellular ligninases in PCP oxidation in PDB cultures, and suggested the role of some intracellular oxidase which has not been documented to date.

### Involvement of induced P450 in PCP transformation

#### In vitro transformation of PCP by microsomal P450

**Figure 3:**
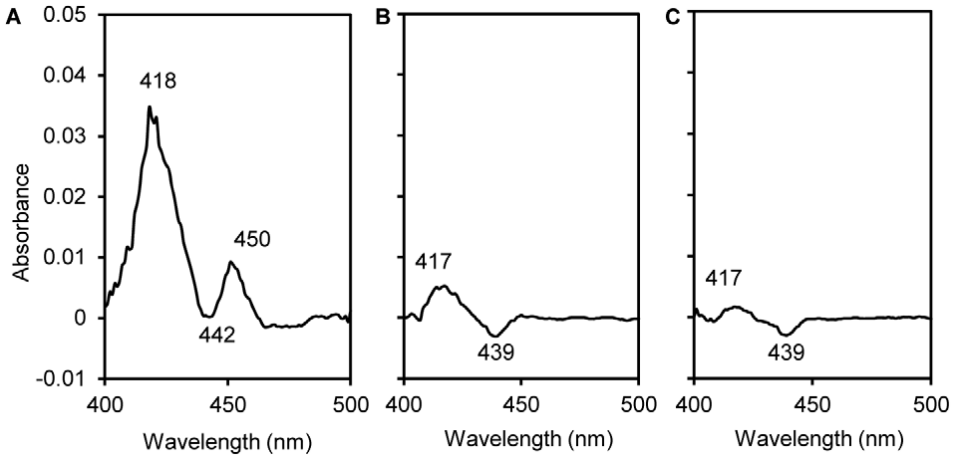
Carbon monoxide difference spectra of microsomal fractions of *Phanerochaete chrysosporium*. The 25-ml potato dextrose broth cultures were added with 37.5 *µ*M pentachlorophenol (dissolved in 25 *µ*l acetone, spectrum A), 25 *µ*l acetone (spectrum B) and without anything (spectrum C), respectively; the protein concentration of every sample tested was 1 mg·ml^−1^.

To demonstrate the existence of P450 during transformation of PCP by P. chrysosporium, CO difference spectra of microsomal fractions from PCP-induced cells were recorded. The spectra of microsomal fractions showed peaks at 450 nm (Figure 3A), indicating the occurrence of P450s, the contents of which were 103±13 pmol P450 per mg protein. The spectra of microsomal fractions had major peaks at 420 nm which were attributed to P420, an inactive form of P450. Because P450 was very slight in pure solvent-induced cells (Figure 3B) and not observed in non-induced cells (Figure 3C), the synthesis of P450 was expected to be induced by addition of PCP to the PDB cultures.

The microsomal fraction with active P450 was utilized for *in vitro* transformation of PCP (Table 1). The microsomal fraction of PCP-induced cells from PDB cultures was utilized after 24-h induction and the concentration of P450 in the reaction mixture was up to 102±13 nM. Samples and controls were prepared as described in Materials and Methods. In the reaction mixture containing P450 and NADPH, PCP transformation was significant (18.8±1.2 nM min^−1^), and 6.5±2.1 *µ*M TCHQ was detected after 10-hour incubation. PB, a P450 inhibitor, was used at 2 mM for inhibition assay. When the mixture was added with PB, PCP transformation rate was decreased by 97% (to 0.6±0.4 nM min^−1^) and TCHQ was not detectable. Furthermore, PCP transformation was not obvious (at an average rate of 0.5±0.4 nM min^−1^) in the mixture with P450 but no NADPH. The reaction mixture without detectable P450 (using microsomal fraction from non-induced cell) and the sterile control had no significant difference in PCP concentration. According to NADPH-dependent PCP transformation, the reaction rate of PCP transformation by the microsomal P450 was up to 19.0±1.2 pmol min^−1^ (mg protein)^−1^.

**Table 1:** Pentachlorophenol (PCP) transformation and concentration of tetrachlorohydroquinone (TCHQ) after incubation of PCP and microsomal fractions of *Phanerochaete chrysosporium* from potato dextrose broth (PDB) cultures.

Initial concentration in reaction mixtures^a^				PCP transformed (*µ*M)^d^	Concentration of TCHQ (*µ*M)	Transformation rate of PCP (nM min^−1^)^d^
PCP (µM)	P450 (nM)	NADPH (mM)	PB (mM)			
37.5	102±13^b^	1	0	11.3±0.7	6.5±2.1	18.8±1.2
37.5	102±13^b^	1	2	0.36±0.24	ND^e^	0.6±0.4
37.5	102±13^b^	0	0	0.30±0.24	ND	0.5±0.4
37.5	ND^c^	1	0	0.10±0.20	ND	0.06±0.12

Mean ± standard deviation of the mean, n = 3

a The reaction mixtures were incubated at 37°C for 10 h

b The microsomal fraction of 37.5 mM PCP-induced cells was utilized.

c The microsomal fraction of non-induced cells was utilized. ND, P450 was not detectable.

d The concentration of PCP transformed and transformation rate of PCP were calculated according to concentrations of residual PCP in the sample and the sterile control after 10-h incubation.

e ND, not detectable (<0.03*µ*M).

#### Effects of the P450 inhibitor and CHI on TCHQ formation in the cultures

The role of P450 was also assessed by examining effect of the P450 inhibitor on PCP transformation in the cultures. We added 2 mM PB to PDB cultures before the addition of 37.5 µM PCP, and TCHQ was not detectable after incubation for 4 days. In contrast, the concentration of TCHQ in the cultures without PB was 0.31±0.09 µM *(31* folds higher than the detection limit) after incubation for 4 days (Table 2). This indicated effective inhibition of TCHQ formation by PB.

**Table 2:** Effect of piperonyl butoxide (PB) and cycloheximide (CHI) on the transformation of pentachlorophenol (PCP) and the production of pentachloroanisole (PCA) and tetrachlorohydroquinone (TCHQ) in potato dextrose broth (PDB) cultures of *Phanerochaete chrysosporium*.

Test condition^a^	PCP transformed (*µ*M)	Concentration of PCA		Concentration of TCHQ		Concentration of cells (g•l^−1^)^c^
		(*µ*M)	(*µ*mol•g^−1^)^b^	(*µ*M)	(*µ*mol•g^−1^)	
Controls	16.9±1.2	15.2±1.2	2.8±0.3	0.31±0.09	0.06±0.02	5.5±0.3
PB	26.4±1.7	21.4±1.0	4.0±0.2	ND^d^	/	5.4±0.2
CHI	17.0±1.0	15.2±1.0	3.4±0.2	ND^d^	/	4.5±0.1

Mean ± standard deviation of the mean, n = 3.

a The 25-ml cultures were incubated for 60 h, and then added with 2 mM PB or 1.46 mM CHI. After another 60-min incubation, the cultures were added with PCP (37.5*µ*M) and incubated for another 4 days before the determination of PCP, PCA and TCHQ by GC-ECD. The cultures without PB or CHI were set as controls. Values represent means ± standard deviations for three replicates.

b *µ*mol•g^−1^, *µ*mol product per gram dry weight of biomass.

c g•l^−1^, gram dry weight of biomass per liter culture.

d ND, not detectable (<0.03 *µ*M).

CHI can suppress *de novo* synthesis of proteins including P450s in the fungi [38]. The PDB cultures was added with 1.46 mM CHI, and then incubated with PCP for 4 days. P450 was not detectable in the microsomal fractions from these cultures. Correspondingly, TCHQ was not detectable either (Table 2). The effects of PB and CHI on TCHQ formation strongly suggesting important role of induced P450 in PCP oxidation.

### Increase of PCA by inhibition of P450

When P450-mediated PCP oxidation was inhibited by PB, the transformation rate of PCP and the concentration of PCA in the cultures were increased by 57±10% and 41±6% (Table 2), and the biomass was not statistically different from the cultures without PB. When induction of P450 was suppressed by CHI, the transformation rate of PCP and the concentration of PCA remained the same (Table 2), but the biomass was decreased by 17±5%. Accordingly, CHI led to increase of PCP transformation and PCA formation in a unit of biomass, by 22±7% and 20±8%. These results suggested interesting relationship between P450 and the methylation.

## Discussion

In our previous work, the induction of P450 by PCP in *P. chrysosporium* was spectrophotometrically detected, but the peaks were at 457∼459 nm in the CO difference spectra [27], [39]. In this paper, the method was modified (e.g., KCN was added to mask cytochrome oxidase before CO difference spectra were recorded). As a result, the typical peak at 450 nm was detected in CO difference spectra of the microsomal fractions of PCP-induced cells, while the peak was not present for non-induced cells and very slight for solvent-induced cells. This result convincingly demonstrated induction of active P450 by PCP.

Induction of P450 by PCP has been reported in algae [40], insect [21] and tissues of many animals, such as human, rat [19] and fish [20]. As for bacteria, induction of a P450-type coenzyme by PCP has only been detected in *Mycobacterium chlorophenolicum*, according to the peak at 457 nm in CO difference spectra [23]. However, induction of fungal P450 by PCP has rarely been documented before, except that of *P. chrysosporium* P450 in our previous paper [39]. The white rot fungus *P. chrysosporium* has about 150 P450 genes [24]. Some of these P450s have been shown to be inducible at the transcriptional level by various xenobiotics [26], [41]–[43], while the effects of chlorophenols on these P450s were not reported. In our work, induction of fungal P450 by PCP was first clarified by the appearance of the typical peak at 450 nm in CO difference spectra.

It is known that fungal P450 systems are involved in oxidation of some xenobiotics, e.g., benzoic acid [33], toluene [44], and phenanthrene [45]. The *P. chrysosporium* P450s co-expressed with P450 oxidoreductase in yeasts exhibited broad substrate profiles [25], [26]. All these eukaryotic P450s, as well as the PCP oxidizing P450 from human [22], utilize NAD(P)H as electron donor of the monooxygenation reactions [46], [47], whereas most bacterial P450s receive electrons from NADH [18]. In the present study, PCP oxidation was considerable in reaction mixture containing microsomal P450 and NADPH, while PCP transformation was not evident in the mixtures without NADPH or sufficient P450, indicating stringent requirement for P450 and NAD(P)H.

The P450-mediated PCP oxidation was further supported by the results of P450 inhibition assay. PB, a well-known inhibitor of P450s, can significantly inhibit many P450-mediated reactions [45], [48], [49], and is expected to have no effect on the total activity of *P. chrysosporium* [50]. In our study, PB significantly inhibited PCP transformation and TCHQ formation in the intact cultures, as well as in the microsomal fractions. Furthermore, CHI which suppressed synthesis of P450 also inhibited the TCHQ formation, and the peroxidases which can produce TCHQ from PCP were not detectable in the cultures. Thus, it is strongly suggested that this fungal P450 could catalyze the oxidation of PCP to form TCHQ in the cultures, which is the same as the P450 in human [22].

According to all the evidences described above, we conclude that P450 is involved in PCP transformation by *P. chrysosporium* and it should be the first time that P450-mediated PCP oxidation was demonstrated in a microorganism. The microsomal P450 of PCP-induced cells catalyzed PCP transformation at a reaction rate of 19.0±1.2 pmol·min^-1^·(mg protein)^−1^ (about 1.1 nmol·h^−1^·[mg protein]^−1^). This is similar to the value of 1.75∼2.2 nmol·h^−1^·(mg protein)^−1^ in the case of the P450-type protein in *M. chlorophenolicum* [23], and higher than 8 pmol·min^−1^·(nmol P450)^−1^ in microsomal fraction of yeast expressing the human P450 34A3 [22].

Laugero et al. [38] reported that mineralization ratio of PCP was up to 11% while that of PCA was only 2% by *P. chrysosporium* under nutrient-rich condition. Moreover, the TCHQ produced by P450-mediated oxidation was expected to be effectively dechlorinated by *P. chrysosporium* according to Reddy and Gold [14]. Thus, the metabolic pathway initiated by P450 was probably an effective way for PCP mineralization other than methylation.

The present work found PCA was the major product of PCP in *P. chrysosporium*, which is consistent with previous studies [3], [38]. PCA was frequently detected in many environments including sediments [51], lake and river waters [52], soils [53] and vegetable materials [54]. Since PCA has a very high bioconcentration factor (10^4^ in fish) and can be rapidly demethylated in both rat and mouse to its more toxic precursor [55], the environmental significant of PCA and other chloroanisoles has been emphasized by several authors [2], [56].

Interestingly, we found obvious increase of PCA in the cultures degrading PCP when P450 was inhibited. PCP methylation was the major source of PCA in environments [57]. P450-mediated demethylations of some chloroanisoles have been reported in animals [58], and a O-demethylation of TCA catalyzed by a microsomal P450 to produce 2,4,6-trichlorophenol has been found in a white rot fungus *Phlebia radiate* recently [56]. Thus, it would be of interest to study on the relationship between P450 and PCP methylation, as well as the role of P450s in PCA metabolism in microorganisms.

## References

[1] Szewczyk R, Długoński J (2009) Pentachlorophenol and spent engine oil degradation by *Mucor ramosissimus* . Int Biodeterior Biodegrad 63: 123–129.

[2] Goswami M, Recio E, Campoy S, Martín JF, Coque JJR (2007) Environmental significance of O-demethylation of chloroanisoles by soil bacterial isolates as a mechanism that improves the overall biodegradation of chlorophenols. Environ Microbiol 9: 2512–2521. 17803776 10.1111/j.1462-2920.2007.01370.x

[3] Walter M, Boul L, Chong R, Ford C (2004) Growth substrate selection and biodegradation of PCP by New Zealand white-rot fungi. J Environ Manage 71: 361–369. 15217724 10.1016/j.jenvman.2004.04.002

[4] Fisher B (1991) Pentachlorophenol: toxicity and environmental fate. J Pestic Reform 11: 2–5.

[5] Gilbert F, Minn C, Duncan R, Wilkinson J (1990) Effects of pentachlorophenol and other chemical preservatives on the health of wood-treating workers in Hawaii. Arch Environ Contam Toxicol 19: 603–609. 2386415 10.1007/BF01059082

[6] Sei K, Takeda T, Soda SO, Fujita M, Ike M (2008) Removal characteristics of endocrine-disrupting chemicals by laccase from white-rot fungi. J Environ Sci Health Part A-Toxic/Hazard Subst Environ Eng 43: 53–60. 10.1080/10934520701750397 18161558

[7] Mahmood S, Paton GI, Prosser JI (2005) Cultivation-independent in situ molecular analysis of bacteria involved in degradation of pentachlorophenol in soil. Environ Microbiol 7: 1349–1360. 16104858 10.1111/j.1462-2920.2005.00822.x

[8] Zhou W, Fu D, Sun Z (1990) A blacklist of priority pollutants in water. Environ Monit in China 6: 1–4.

[9] Rubilar O, Tortella G, Cea M, Acevedo F, Bustamante M, et al (2011) Bioremediation of a Chilean Andisol contaminated with pentachlorophenol (PCP) by solid substrate cultures of white-rot fungi. Biodegradation 22: 31–41. 20512655 10.1007/s10532-010-9373-9

[10] Yu Z, Zeng GM, Chen YN, Zhang JC, Yu Y, et al (2011) Effects of inoculation with Phanerochaete chrysosporium on remediation of pentachlorophenol-contaminated soil waste by composting. Process Biochemistry 46: 1285–1291.

[11] Pedroza AM, Mosqueda R, Alonso-Vante N, Rodriguez-Vazquez R (2007) Sequential treatment via Trametes versicolor and UV/TiO2/RuxSey to reduce contaminants in waste water resulting from the bleaching process during paper production. Chemosphere 67: 793–801. 17123583 10.1016/j.chemosphere.2006.10.015

[12] Xun L, Orser CS (1991) Purification and properties of pentachlorophenol hydroxylase, a flavoprotein from *Flavobacterium* sp. strain ATCC 39723. J Bacteriol 173: 4447–4453. 2066340 10.1128/jb.173.14.4447-4453.1991 PMC208108

[13] Peng D, Xie G-X, Zeng G-M, Chen Y-N, Chen F-R, et al (2008) Research and application of producing laccase by *Phanerochaete chrysosporium* in solid-state fermentation system. Environ Sci 29: 3568–3573. 19256402

[14] Reddy GVB, Gold MH (2000) Degradation of pentachlorophenol by *Phanerochaete chrysosporium*: intermediates and reactions involved. Microbiology-(UK) 146: 405–413. 10.1099/00221287-146-2-405 10708379

[15] Zhang JB, Ye P, Chen S, Wang WJ (2007) Removal of pentachlorophenol by immobilized horseradish peroxidase. Int Biodeterior Biodegrad 59: 307–314.

[16] Neilson AH, Lindgren C, Hynning PA, Remberger M (1988) Methylation of halogenated phenols and thiophenols by cell extracts of Gram-positive and Gram-negative bacteria. Appl Environ Microbiol 54: 524–530. 16347565 10.1128/aem.54.2.524-530.1988 PMC202484

[17] Machado KMG, Matheus DR, Monteiro RTR, Bononi VLR (2005) Biodegradation of pentachorophenol by tropical basidiomycetes in soils contaminated with industrial residues. World J Microbiol Biotechnol 21: 297–301.

[18] Omura T (1999) Forty years of cytochrome P450. Biochem Biophys Res Commun 266: 690–698. 10603307 10.1006/bbrc.1999.1887

[19] Dubois M, Grosse Y, Thome JP, Kremers P, PfohlLeszkowicz A (1997) Metabolic activation and DNA-adducts detection as biomarkers of chlorinated pesticide exposures. Biomarkers 2: 17–24. 23899150 10.1080/135475097231922

[20] Livingstone DR (1998) The fate of organic xenobiotics in aquatic ecosystems: quantitative and qualitative differences in biotransformation by invertebrates and fish. Comp Biochem Physiol A-Mol Integr Physiol 120: 43–49. 9773498 10.1016/s1095-6433(98)10008-9

[21] Suwanchaichinda C, Brattsten LB (2001) Effects of exposure to pesticides on carbaryl toxicity and cytochrome P450 activities in *Aedes albopictus* larvae (Diptera : Culicidae). Pest Biochem Physiol 70: 63–73.

[22] Mehmood Z, Williamson MP, Kelly DE, Kelly SL (1996) Metabolism of organochlorine pesticides: the role of human cytochrome P450 3A4. Chemosphere 33: 759–769. 8759309 10.1016/0045-6535(96)00212-3

[23] Uotila JS, Salkinoja-Salonen MS, Apajalahti JH (1991) Dechlorination of pentachlorophenol by membrane bound enzymes of *Rhodococcus chlorophenolicus* PCP-I. Biodegradation 2: 25–31. 1368474 10.1007/BF00122422

[24] Martinez D, Larrondo LF, Putnam N, Gelpke MDS, Huang K, et al (2004) Genome sequence of the lignocellulose degrading fungus *Phanerochaete chrysosporium* strain RP78. Nat Biotechnol 22: 695–700. 15122302 10.1038/nbt967

[25] Hirosue S, Tazaki M, Hiratsuka N, Yanai S, Kabumoto H, et al (2011) Insight into functional diversity of cytochrome P450 in the white-rot basidiomycete Phanerochaete chrysosporium: Involvement of versatile monooxygenase. Biochem Biophys Res Commun 407: 118–123. 21362401 10.1016/j.bbrc.2011.02.121

[26] Syed K, Doddapaneni H, Subramanian V, Lam YW, Yadav JS (2010) Genome-to-function characterization of novel fungal P450 monooxygenases oxidizing polycyclic aromatic hydrocarbons (PAHs). Biochem Biophys Res Commun 399: 492–497. 20674550 10.1016/j.bbrc.2010.07.094 PMC2943217

[27] Ning DL, Wang H, Wang LH, Ding C (2009) Induction and function of cytochrome P450 in degradation of refractory organic chemicals by *Phanerochaete chrysosporium*. China Environ Sci: 407–412.

[28] Aiken BS, Logan BE (1996) Degradation of pentachlorophenol by the white rot fungus *Phanerochaete chrysosporium* grown in ammonium lignosulphonate media. Biodegradation 7: 175–182. 8782389 10.1007/BF00058177

[29] Reddy GVB, Gelpke MDS, Gold MH (1998) Degradation of 2,4,6-trichlorophenol by *Phanerochaete chrysosporium*: Involvement of reductive dechlorination. J Bacteriol 180: 5159–5164. 9748450 10.1128/jb.180.19.5159-5164.1998 PMC107553

[30] Masaphy S, Levanon D, Henis Y, Venkateswarlu K, Kelly SL (1996) Evidence for cytochrome P-450 and P-450-mediated benzo(a)pyrene hydroxylation in the white rot fungus *Phanerochaete chrysosporium* . FEMS Microbiol Lett 135: 51–55. 8598277 10.1111/j.1574-6968.1996.tb07965.x

[31] Ning DL, Wang H, Li D (2009) Induction and measurement of cytochrome P450 in white rot fungi. Environ Sci 30: 2485–2490. 19799321

[32] Omura T, Sato R (1964) The carbon monoxide-binding pigment of liver microsomes. J Biol Chem 239: 2370–2378. 14209971

[33] Ning DL, Wang H, Zhuang Y (2010) Induction of functional cytochrome P450 and its involvement in degradation of benzoic acid by *Phanerochaete chrysosporium* Biodegradation 21.. 10.1007/s10532-009-9301-z 19787435

[34] Bradford MM (1976) A rapid and sensitive method for the quantification of microgram quantities of protein utilizing the principle of protein-dye binding. Anal Biochem 72: 248–254. 942051 10.1016/0003-2697(76)90527-3

[35] Tien M, Kirk TK (1988) Lignin peroxidase of *Phanerochaete chrysosporium* . Methods Enzymol 161: 238–249.

[36] Paszczynski A, Crawford RL, Huynh VB (1988) Manganese peroxidase of *Phanerochaete chrysosporium*: Purification. Methods Enzymol 161: 264–270.

[37] Niku-Paavola ML, Karhunen E, Salola P, Raunio V (1988) Ligninolytic enzymes of the white-rot fungus *Phlebia radiata* . Biochem J 254: 877–884. 3196301 10.1042/bj2540877 PMC1135164

[38] Laugero C, Mougin C, Sigoillot JC, Moukha S, Asther M (1997) Comparison of static and agitated immobilized cultures of *Phanerochaete chrysosporium* for the degradation of pentachlorophenol and its metabolite pentachloroanisole. Can J Microbiol 43: 378–383.

[39] Ning DL, Wang H, Zhuang Y, Li D (2007) Inducement and degradation function of p450 in *Phanerochaete chrysospofium* by *in situ* spectroscopic analysis. Chem J Chin Univ-Chin 28: 1469–1474.

[40] Barque JP, Abahamid A, Flinois JP, Beaune P, Bonaly J (2002) Constitutive overexpression of immunoidentical forms of PCP-induced *Euglena gracilis* CYP-450. Biochem Biophys Res Commun 298: 277–281. 12387828 10.1016/s0006-291x(02)02439-7

[41] Chigu NL, Hirosue S, Nakamura C, Teramoto H, Ichinose H, et al (2010) Cytochrome P450 monooxygenases involved in anthracene metabolism by the white-rot basidiomycete Phanerochaete chrysosporium. Appl Microbiol Biotechnol 87: 1907–1916. 20508934 10.1007/s00253-010-2616-1

[42] Doddapaneni H, Yadav JS (2005) Microarray-based global differential expression profiling of P450 monooxygenases and regulatory proteins for signal transduction pathways in the white rot fungus *Phanerochaete chrysosporium* . Mol Genet Genomics 274: 454–466. 16231151 10.1007/s00438-005-0051-2

[43] Subramanian V, Yadav JS (2009) Role of P450 Monooxygenases in the Degradation of the Endocrine-Disrupting Chemical Nonylphenol by the White Rot Fungus Phanerochaete chrysosporium. Appl Environ Microbiol 75: 5570–5580. 19542331 10.1128/AEM.02942-08 PMC2737932

[44] Luykx DMAM, Prenafeta-Boldu FX, de Bont JAM (2003) Toluene monooxygenase from the fungus *Cladosporium sphaerospermum* . Biochem Biophys Res Commun 312: 373–379. 14637148 10.1016/j.bbrc.2003.10.128

[45] Ning DL, Wang H, Ding C, Lu HJ (2010) Novel evidence of cytochrome P450-catalyzed oxidation of phenanthrene in *Phanerochaete chrysosporium* under ligninolytic conditions. Biodegradation 21: 889–901. 20333538 10.1007/s10532-010-9349-9

[46] Ichinose H, Wariishi H (2012) Heterologous expression and mechanistic investigation of a fungal cytochrome P450 (CYP5150A2): Involvement of alternative redox partners. Arch Biochem Biophys 518: 8–15. 22206618 10.1016/j.abb.2011.12.010

[47] Syed K, Kattamurib C, Thompsonb TB, Yadav JS (2011) Cytochrome b5 reductase–cytochrome b5 as an active P450 redox enzyme system in Phanerochaete chrysosporium: A typical properties and in vivo evidence of electron transfer capability to CYP63A2. Arch Biochem Biophys 509: 26–32. 21376009 10.1016/j.abb.2011.02.023 PMC3119499

[48] Kamada F, Abe S, Hiratsuka N, Wariishi H, Tanaka H (2002) Mineralization of aromatic compounds by brown-rot basidiomycetes - mechanisms involved in initial attack on the aromatic ring. Microbiology-(UK) 148: 1939–1946. 10.1099/00221287-148-6-1939 12055313

[49] Murray M, Reidy GF (1990) Selectivity in the inhibition of mammalian cytochromes P450 by chemical agents. Pharmacol Rev 42: 85–101. 2198606

[50] Teramoto H, Tanaka H, Wariishi H (2004) Degradation of 4-nitrophenol by the lignin-degrading basidiomycete *Phanerochaete chrysosporium* . Appl Microbiol Biotechnol 66: 312–317. 15448939 10.1007/s00253-004-1637-z

[51] Barakat AO, Moonkoo K, Yoarong Q, Wade TL (2002) Organochlorine pesticides and PCB residues in sediments of Alexandria Harbour, Egypt. Mar Pollut Bull 44: 1426–1434. 12523549 10.1016/s0025-326x(02)00313-2

[52] Jiang X, Martens D, Schramm KW, Kettrup A, Xu SF, et al (2000) Polychlorinated organic compounds (PCOCs) in waters, suspended solids and sediments of the Yangtse River. Chemosphere 41: 901–905. 10864163 10.1016/s0045-6535(99)00435-x

[53] Palm H, Knuutinen J, Haimi J, Salminen J, Huhta V (1991) Methylation products of chlorophenols, catechols and hydroquinones in soil and earthworms of sawmill environments. Chemosphere 23: 263–267.

[54] McLellan I, Carvalho M, Pereira CS, Hursthouse A, Morrison C, et al (2007) The environmental behaviour of polychlorinated phenols and its relevance to cork forest ecosystem: a review. J Environ Monitor 9: 1055–1063. 10.1039/b701436h 17909638

[55] Yuan JH, Goehl TJ, Murrill E, Moore R, Clark J, et al (1993) Toxicokinetics of pentachloroanisole in F344 rats and B6C3F1 mice. Xenobiotica 23: 427–438. 8337901 10.3109/00498259309057031

[56] Campoy S, Alvarez-Rodriguez ML, Recio E, Rumbero A, Coque JJR (2009) Biodegradation of 2,4,6-TCA by the white-rot fungus *Phlebia radiata* is initiated by a phase I (O-demethylation)-phase II (O-conjugation) reactions system: implications for the chlorine cycle. Environ Microbiol 11: 99–110. 18783381 10.1111/j.1462-2920.2008.01744.x

[57] Wittlinger R, Ballschmiter K (1990) Studies of the global baseline pollution, XIII. Fresenius J Anal Chem 336: 193–200.

[58] Miller GP, Guengerich FP (1999) Anisole demethylation by P450 1A2: Binding and steady-state studies. Faseb J 13: A1346–A1346.

